# Deep Learning and Radiomics predict complete response after neo-adjuvant chemoradiation for locally advanced rectal cancer

**DOI:** 10.1038/s41598-018-30657-6

**Published:** 2018-08-22

**Authors:** Jean-Emmanuel Bibault, Philippe Giraud, Martin Housset, Catherine Durdux, Julien Taieb, Anne Berger, Romain Coriat, Stanislas Chaussade, Bertrand Dousset, Bernard Nordlinger, Anita Burgun

**Affiliations:** 1 Radiation Oncology Department, Georges Pompidou European Hospital, Assistance Publique – Hôpitaux de Paris, Paris Descartes University, Paris Sorbonne Cité, Paris, France; 2 INSERM UMR 1138 Team 22: Information Sciences to support Personalized Medicine, Paris Descartes University, Sorbonne Paris Cité, Paris, France; 3 Department of gastroenterology and digestive oncology, Georges Pompidou European Hospital, Assistance Publique – Hôpitaux de Paris, Paris Descartes University, Paris Sorbonne Cité, Paris, France; 4 Department of General Surgery and Surgical Oncology, Georges Pompidou European Hospital, Assistance Publique – Hôpitaux de Paris, Paris Descartes University, Paris Sorbonne Cité, Paris, France; 5 Gastroenterology and digestive oncology unit, Cochin University Hospital, Assistance Publique – Hôpitaux de Paris, Paris, France; 6 INSERM Y 1016, Université Paris Descartes, Paris, France; 7 Department of Digestive, Hepato-biliary and Endocrine Surgery, Cochin Hospital, Assistance Publique – Hôpitaux de Paris, Paris, France; 8 Department of General Surgery and Surgical Oncology, Hôpital Ambroise Paré, Assistance Publique – Hôpitaux de Paris, Boulogne-Billancourt, France; 9 Biomedical Informatics and Public Health Department, Georges Pompidou European Hospital, Assistance Publique – Hôpitaux de Paris, Paris Descartes University, Paris Sorbonne Cité, Paris, France

## Abstract

Treatment of locally advanced rectal cancer involves chemoradiation, followed by total mesorectum excision. Complete response after chemoradiation is an accurate surrogate for long-term local control. Predicting complete response from pre-treatment features could represent a major step towards conservative treatment. Patients with a T2-4 N0-1 rectal adenocarcinoma treated between June 2010 and October 2016 with neo-adjuvant chemoradiation from three academic institutions were included. All clinical and treatment data was integrated in our clinical data warehouse, from which we extracted the features. Radiomics features were extracted from the tumor volume from the treatment planning CT Scan. A Deep Neural Network (DNN) was created to predict complete response, as a methodological proof-of-principle. The results were compared to a baseline Linear Regression model using only the TNM stage as a predictor and a second model created with Support Vector Machine on the same features used in the DNN. Ninety-five patients were included in the final analysis. There were 49 males (52%) and 46 females (48%). Median tumour size was 48 mm (15–130). Twenty-two patients (23%) had pathologic complete response after chemoradiation. One thousand six hundred eighty-three radiomics features were extracted. The DNN predicted complete response with an 80% accuracy, which was better than the Linear Regression model (69.5%) and the SVM model (71.58%). Our model correctly predicted complete response after neo-adjuvant rectal chemoradiotherapy in 80% of the patients of this multicenter cohort. Our results may help to identify patients who would benefit from a conservative treatment, rather than a radical resection.

## Introduction

The standard treatment for locally advanced rectal cancer is neoadjuvant chemoradiation followed by total mesorectal excision (TME) after a 6 to 10 week interval^1^. In 20% to 30% of the patients, no residual tumor is found at histopathology^2^. In this selected group of patients, it has been suggested that surgery could be omitted, since it did not improve outcome and was associated with a high rate of morbidity^3^. This strategy has initially been met with significant skepticism, but organ preservation strategies with watch-and-wait or transanal endoscopic microsurgery have since shown good long-term results^2,4–8^.

Complete pathologic response (pCR) to neo-adjuvant chemoradiation is assessed during pathological examination after surgery. Identifying patients in pCR with a high rate of accuracy could lead to improved clinical outcome. Computational Imaging, also known as Radiomics, is the use of imaging data from routine clinical work-up to assess the tumor characteristics, such as spatial heterogeneity, texture or shape. This approach is transforming imaging into a high-throughput data mine that can be leveraged and analyzed with other clinical features for precision medicine and decision support. Its potential is currently being explored in several clinical setups^9^, including rectal cancer^10^.

Deep Learning (DL) is a subfield of machine learning and artificial intelligence that is increasingly used in medicine^11–14^ for diagnosis^15^, classification^16^, or prediction^17,18^. In this study, we present a novel approach combining Deep Learning with clinical and radiomics features to build a model predicting pCR in a multicenter cohort of patients with locally-advanced rectal cancer treated with neo-adjuvant chemoradiation, followed by surgery.

## Results

### Radiomics features

**Figure 1:**
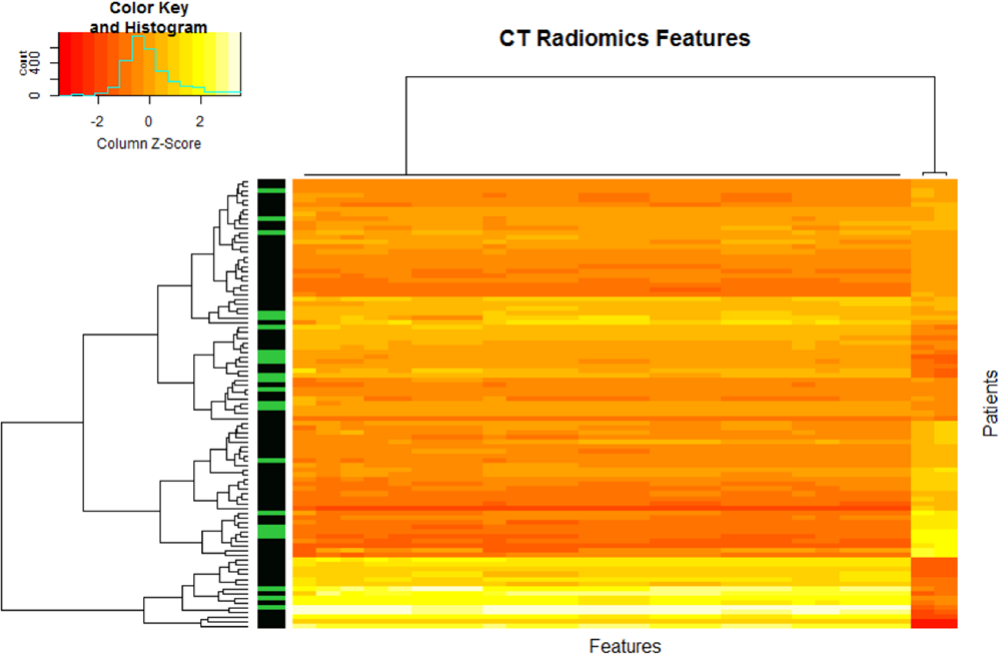
Heatmap of radiomics features correlated to complete response.

One thousand six hundred eighty-three features were extracted from the two segmentations of the tumor volume, for each patient (319770 features in total). One hundred and twenty-four features (7.3%) had an Intraclass Correlation Coefficient higher than 0.8 in the following categories: Texture (Grey-Level Co-Occurrence Matrix in 2D and 3D, Grey-Level Run Length Matrix, Intensity Direct and Intensity Histogram) and Shape. Out of these, 28 features (22%) were filtered on the basis that they were significantly correlated to pathological Complete Response (Wilcoxon Test p < 0.05) in three categories of features: Gray-Level Co-ocurrence Matrix 2D and 3D and IntensityDirect. The heatmap showed clustering of these features in two groups of patients (Fig. 1).

### Deep Learning network training and testing

As a baseline, another model created with Linear Regression, using only the TNM stage, showed a lower accuracy of 69,5% (95% CI = 59,2% to 78,51%). Sensitivity was 34,78% (95%CI = 16,38% to 57.27%), specificity was 80,56% (95% CI = 69.53% to 88.94%). Mean area under the curve (AUC) for the LR model was 0.59 (95% CI = 0.46 to 0.69). In the DNN, 29 variables (T stage and the robust, filtered 28 radiomics features) were included. The DNNClassifier predicted pCR with an 80% accuracy (95% CI = 70.54% to 87.51%). Sensitivity was 68.2% (95%CI = 45.13% to 86.14%), specificity was 83.56% (95% CI = 73.05% to 91.21%). Mean AUC for the DNN model was 0.72 (95% CI = 0.65 to 0.87). A comparison of the main metrics of the model (accuracy, AUC, false and true positives and negatives rates) did not reveal any significant differences in the results between the folds (Chi-Squared test, p > 0.05). Increasing the number of hidden layers or the neurons in each layer did not improve performance: accuracy dropped to 70% when ten hidden layers where used with 100 neurons in each layer. A ten-fold increase of the number of learning steps (n = 30,000) or epochs (n = 10) for network training did not improve accuracy (80%).

As a comparison, a Support Vector Machine (SVM) model was created. The accuracy of this model, trained on the same features with a 5-fold cross validation, was 71.58% (95% CI = 61.40% to 80.36%). Sensitivity was 45.45% (95% CI = 24,39% to 67,79%), specificity was 79.45% (95% CI = 68.38% to 88.02%). Mean AUC for the SVM model was 0.62 (95% CI = 0.51 to 0.74). Confusion matrices for the LR, DNN and SVM models are shown in Table 1. There was no statistical difference for the metrics of the model between the fold (Chi-Squared test, p > 0.05).

**Table 1:** Confusion matrices: Baseline LR: Linear Regression model with T stage; DNN: Deep Neural Network model with 29 variables); SVM: Support Vector Machine Model with the same 29 variables.

		Predicted class		
		pCR: n (% of actual class)	Non pCR: n (% of actual class)	Total
Actual class	pCR	Baseline LR: 8 (36%)	Baseline LR: 14 (64%)	22
		DNN: 15 (68%)	DNN: 7 (32%)	
		SVM: 10 (45,4%)	SVM: 12 (54,5%)	
	Non pCR	Baseline LR: 15 (21%)	Baseline LR: 58 (79%)	73
		DNN: 12 (16%)	DNN: 61 (84%)	
		SVM: 15 20%)	SVM: 62 (80%)	
	Total	LR: 23	LR: 72	95
		DNN: 27	DNN: 68	
		SVM: 25	SVM: 70	

**Figure 2:**
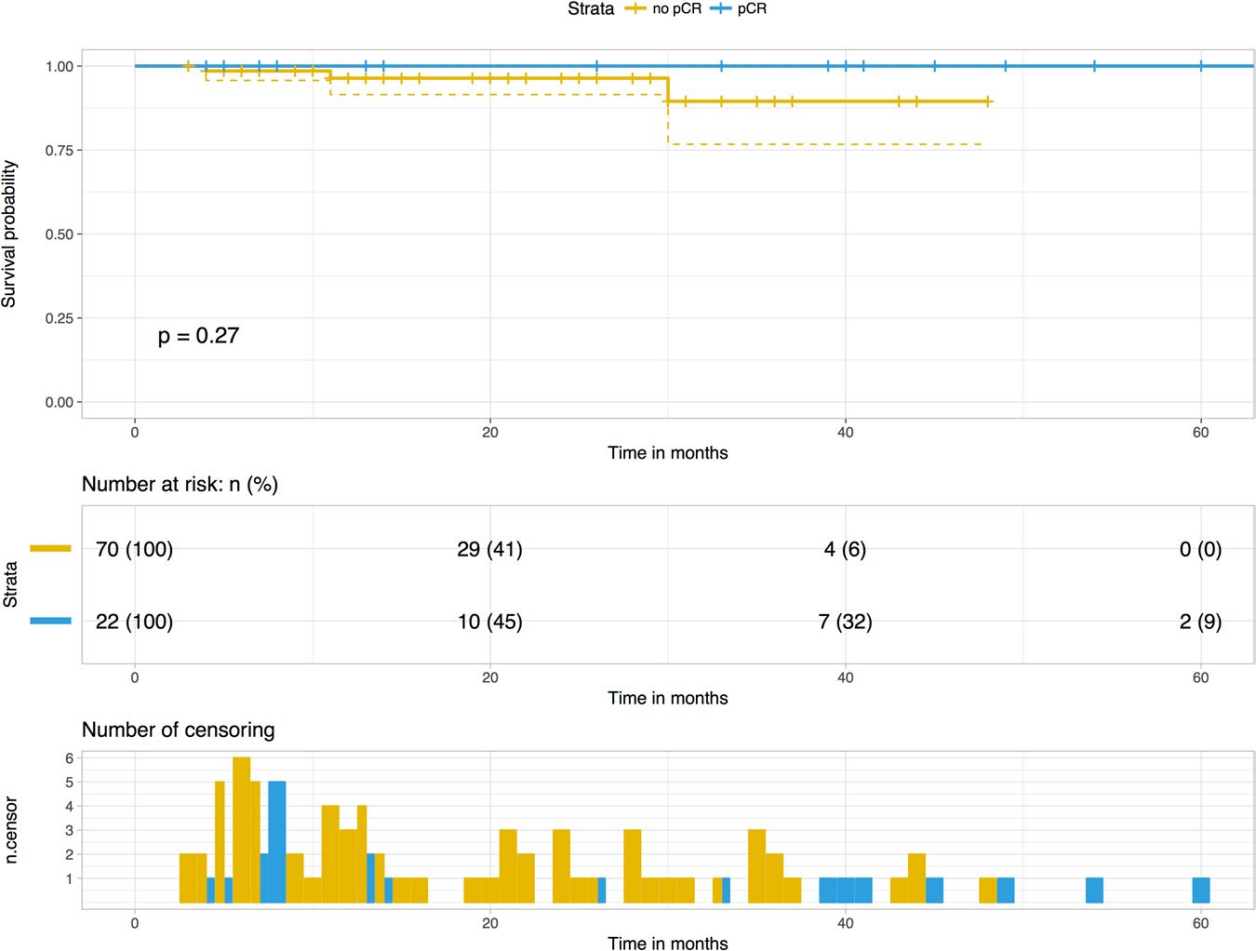
Kaplan-Meier curves for overall survival stratified on pathological complete response.

There was no statistical correlation between pCR and overall survival (log-rank test, p = 0.258), but none of the patients in pCR died during the study (Fig. 2).

## Discussion

This is the first study that used Deep Learning to predict pCR after neo-adjuvant chemoradiation in locally advanced rectal cancer. Routine care data were extracted from our CDW to create a profile of the patients. T stage was the only variable significantly correlated to pCR. It was combined with a high-dimensional radiomics phenotype, extracted from CT scans, in a Deep Neural Network to predict pCR after neoadjuvant chemoradiation. The DNN predicted pCR correctly in 80% of the cases. Creating a profile of each patient was made possible through the use of our CDW, in which medical records of all our patients are prospectively and automatically stored. Data from 750,000 patients are stored in the HEGP CDW, including 14,000 cancer patients treated with radiation. A request can easily be created in i2b2 to identify cohorts of patients that can later be used to extract structured and unstructured data from the CDW, using custom-made software^19^. In the field of radiation oncology, data from treatment planning and delivery can also be easily extracted from the Treatment Planning and Record-and-Verify systems.

Computational Imaging consists in extracting quantitative features from CT scan, MRI or PET/CT^20^. Medical images are no longer simple pictures to interpret visually; they are now treated as data. These analyses are intended to be conducted with routine care images and could be used like any other data for target volume delineation or decision-making^21^. However, there are several tools available and no standard way to extract radiomics features, meaning that reproducibility is a key challenge in this field^22^. We used an open and free tool that was designed for collaboration^23^. We provide the parameters we used for feature extraction and selection in Supplementary File 1 and online^24^. Robustness of the extracted features was assessed with ICC computation, and only features with a high ICC (>0.8) were kept.

Other studies have already been published, using MRI^25^ or PET/CT^26^ with logistic regression or simpler Artificial Neural Network (ANN) to predict treatment response, with correct accuracy (AUC = 0.71–0.79 for the MRI model, data not provided for the PET/CT model). The latest study included 222 patients to build a radiomics signature with 30 MRI features^25^. The model was created with Support Vector Machine, another Machine Learning approach^13^, and found an area under the receiver operating characteristic curve of 0.9756 (95% confidence interval, 0.9185–0.9711) in the validation cohort. However, feature extraction from MRI is even more complex and less reproducible than CT Scan, meaning that these results cannot be easily reproduced on another cohort^27^. Another frequent limit of radiomics studies is that they often explore the prognostic relevance of imaging features, without using any clinical, biological or treatment delivery data. This was not the case in our study since we used several other inputs beyond radiomics into our DNN. Our study has some limitations. First, the sample size is limited with a large number of predictors. In our cohort, patients were referred from three different hospitals for neoadjuvant chemoradiation and were then treated in their respective institution, which limits the risk of overfitting (i.e. when a neural network has been trained on a dataset and is unable to accurately predict the outcome on another unknown dataset). A 5-fold cross validation was performed. The feature selection performed before training could have caused some test-set leakage. To limit this, we selected features from 4 of the 5 partitioned datasets, leaving the validation set from the first validation. Since the accuracy and AUC of the model is consistent across all five cross-validations, we do not believe leakage had a significant effect on the model.

Physicians cannot intuitively understand the results given by the model we created: Deep Learning essentially remains a black box. With the use of a large number of variables and second order statistical data, such as radiomics, we believe this cannot be avoided. Visualization techniques such as a radiomics heatmap can only give a high-level representation of the data. These publications are hypothesis-generating studies and can help in identifying relevant prognostic or predictive factors, but their level of evidence is still low, no matter how innovative their approach is. It is considered that a human brain can only integrate up to 5 variables in order to make an adequate decision^28,29^. Since oncology is relying on an increasing amount of data of different types, using computers as Clinical Decision Support Systems (CDSS) could become mandatory. Deep Learning, and Artificial Intelligence in a broader sense, will eventually disrupt the way we practice medicine in positive^11,30,31^ and negative^32^ ways. Among the disciplines poised to be radically changed, is medical imaging. Several studies have recently been published predicting longevity from routine CT-Scans^18^ or detecting pneumonia from chest X-Ray^33^. The development of Deep Learning will transform the way we use imaging for diagnosis, treatment planning and decision making. It is not clear yet if these methods should be assessed as any other medical device in a randomized trial or if new approaches are needed.

## Conclusion

In this proof-of-concept study, we show that using a DNNClassifier on heterogeneous data combining clinical and radiomics features is feasible and can accurately predict patients who will have a complete pathological response after neo-adjuvant chemoradiotherapy for locally-advanced rectal cancer. For this subset of patients, conservative treatments could be a valid approach, with less long-term side effects. After careful prospective evaluation of this approach in a randomized clinical trial, this kind of methods could be directly implemented within the treatment planning systems used in radiation oncology to better personalize treatments.

## Methods

### Ethical statement

This study was approved by the IRB and ethics committee CPP Ile-de-France II: IRB Committee # 00001072, study reference # CDW_2015_0024. All experiments were carried out in accordance with relevant guidelines and regulations.

The study used only pre-existing medical data, therefore patient consent was not required by the ethics committee.

### Dataset description

Patients with a T2-4 N0-1 rectal adenocarcinoma treated between June 2010 and October 2016 with neo-adjuvant chemoradiation (4 to 50.4 Gy) with Capecitabine (800 mg/m^2^ twice a day) were included in the study. The patient recruitment originated from three academic institutions: Hôpital Européen Georges Pompidou (HEGP), Hôpital Cochin (HC) and Hôpital Ambroise Paré (HAP), belonging to the Assistance Publique – Hôpitaux de Paris. All patients had a pelvic MRI and PET-CT for staging. Chemoradiation was performed in our department and surgery was performed later 6 to 10 weeks by the surgery department in each recruiting institution. Ninety-five patients from three different institutions (HEGP: n = 35, 37%; HC: n = 23, 24%; HAP: n = 37, 39%) were included in the final analysis. Median follow-up was 16 months (range: 3–65). There were 49 males (52%) and 46 females (48%). Median age was 66 years old (32–84). Median tumor size was 48 mm (15–130). There were 9 T2 (9%), 75 T3 (79%) and 11 T4 (12%) tumors. Nineteen patients (20%) had no lymph node metastasis on pelvic MRI and there were 76 N+ patients (80%). Median baseline hemoglobin, neutrophils and lymphocytes counts were 13.6 g/dl (9.7–17.5), 1734/mm^3^ (336–3760) and 4050/mm^3^ (1100–11160) respectively. Delivered median doses were 50.4 Gy (45–50.4) to the GTVp and 45 Gy to the CTV. Median dose per fraction was 2 Gy (1.8–2.25 Gy). Median treatment length was 39 days (32–69). Average time between chemoradiation and surgery was 9 weeks (min = 4; max = 11). Twenty-two patients (23%) had pathologic complete response after chemoradiation. Forty-two patients received adjuvant chemotherapy. Patients’ characteristics from each institution are shown in Table 2. Two patients had a local relapse (2.1%, none in the pCR group) and 7 a distant relapse (7.3%, none in the pCR group) during follow-up. Disease-free and overall survival rates at follow-up were 90.53% and 96.85% respectively. Among the variables extracted from our CDW, T stage was the only feature significantly correlated with pCR (Chi-Squared test, p = 0.036).

**Table 2:** Patient characteristics.

Characteristics	HEGP (n = 35–37%)	HC (n = 23–24%)	HAP (n = 37–39%)	Chi-Squared Test (p)
**Sex**				
Male	18 (51.4%)	14 (61%)	17 (54%)	0.603
Female	17 (48.6%)	9 (39%)	20 (46%)	
Median age (range)	65 (34–79)	61 (37–84)	70 (32–84)	0.768
**T stage**				
2	2 (5.7%)	0 (0%)	7 (19%)	0.587
3	27 (77.2%)	20 (87%)	28 (75.6%)	
4	6 (17.1%)	3 (13%)	2 (5.4%)	
**N stage**				
0	9 (25.4%)	4 (17.4%)	6 (16%)	0.588
1	26 (74.6%)	19 (82.6%)	31 (84%)	
Median tumor size in mm (range)	50 (15–130)	48 (30–65)	45 (20–99)	0.954
**Tumor differentiation**				
Grade 1	0 (0%)`	0 (0%)	0 (0%)	0.118
Grade 2	0 (0%)	2 (8.7%)	1 (2%)	
Grade 3	16	12 (52.2%)	16 (44%)	
Grade 4	19	9 (39.1%)	20 (54%)	
Median baseline hemoglobin in g/dl (range)	13.3 (9.7–15.5)	13.6 (11.3–17.5)	13.5 (10.3–17)	0.220
Median baseline neutrophils in /mm^3^ (range)	3659 (1100–9380)	3879 (1820–10925)	3796 (1740–11160)	0.405
Median baseline lymphocytes in /mm^3^ (range)	1638 (336–3504)	1740 (1159–3240)	1844 (520–3760)	0.327
Median dose to GTVp in Gy (range)	50.4 (45–50.4)	50 (45–50.4)	50 (45–50.4)	0.127
Median dose to CTV in Gy (range)	45 (45–46)	46 (45–49.5)	46 (45–46)	0.204
Median dose per fraction in Gy (range)	1.8 (1.8–2)	2 (1.8–2.25)	2 (1.8–2)	0.001
Median treatment length in days (range)	48 (32–54)	37 (32–69)	37 (32–113)	0.181
Pathological complete response rate (n - %)	9 (25.7%)	3 (12.5%)	10 (27%)	0.387

### Clinical, pathological, biological features extraction

All features were extracted from our clinical data warehouse (CDW), that relies on the Informatics for Integrating Biology and the Bedside (i2b2) model - an open source infrastructure developed by Harvard Medical School and adopted by more than 130 academic hospitals around the world^34,35^. The i2b2 warehouse uses an Entity-Attribute-Value (EAV) data model for its adaptability and dynamic nature. Concepts are stored separately in a hierarchical data model. The following features were extracted: age, sex, smoking status, tumor differentiation and size, T and N stages, baseline hemoglobin, neutrophils and lymphocytes counts.

### Treatment planning and delivery features extraction

Data on treatment planning and delivery in our institution (2001–2016) were extracted from the ARIA system using reverse engineering and the VARIAN ESAPI^36^ for dose-volume histograms (DVH). Structures labels were sorted and filtered by number of occurrences. Each of them was then matched to the ROS ontology before integration into the CDW^37–39^. Delivered total dose to the GTVp and CTV, dose per fraction and treatment length were then extracted for modeling.

### Radiomics features extraction, quality control and filtering

All included patients’ treatment planning CT-Scans were extracted from the institution PACS and reused. CT-Scan were performed on scanned on a General Electric Light Speed scanner (Boston, Massachusetts, USA) as follow: helical acquisition, contiguous slices of 1.25 mm, 512 × 512 × 512 matrix, 120 kV, mAs > 350, speed: 3.75, mode: 0.75, with contrast injection, software version 0.7MW11.10. The rectal Primary Gross Tumor Volume (GTVp) was manually segmented by two expert gastrointestinal radiation oncologists on Eclipse V.13 (Varian, Palo Alto, California, USA) before being exported for radiomics features extraction. All DICOM and RT-STRUCT data were imported into IBEX (MD Anderson, Texas, USA), an open infrastructure platform dedicated to radiomics features extraction^23^. Each patient was segmented twice. Shape, Intensity Direct, Gray Level Co-Ocurrence Matrix (GLCM) 25 (computed from all 2D image slices) and 3 (computed from all 3D image matrices), Neighbor Intensity Difference (NID) 3 and 25, Gray Level Run Length Matrix 25 were extracted without pre-processing on each segmentation. In all, 319 770 features were extracted (1683 features extracted for each segmentation on each 95 patients). All extraction categories are provided (Supplementary File 1). To estimate the robustness of the tumor features, the intra-class correlation coefficient (ICC) was calculated)^40,41^. ICC can be used when quantitative measurements are made on units that are organized into groups^42^. It ranges between 0 and 1, indicating null and perfect reproducibility. In order to determine the ICC (equation 1) for inter-observer segmentations, variance estimates were obtained from two-way mixed effect model of analysis of variance (ANOVA):

1 $$ICC=\frac{MSr-MSw}{MSr+(k-1)MSw}$$

where MSR = mean square for rows, MSW = mean square for residual source of variance, k = number of observers involved and n = number of subjects. R version 3.4.2 with the ICC package was used for computation^43^.

### Statistical analysis

Chi-squared tests were performed to evaluate differences in patients’ characteristics between institutions and to select categorical variables correlated to pCR (p < 0.05). Wilcoxon tests were performed to filter features significantly correlated (p < 0.05) with pCR. As Parmar *et al*. showed, Wilcoxon test-based feature selection has the highest prognostic performance with high stability against data perturbation^44^. A heatmap showing radiomics features clustering and correlations to pCR was generated. Survival rates were calculated from the date of surgery to create a Kaplan-Meier curve for overall survival. All statistical analysis were performed with R version 3.4.2^45^ with the ggplot2^46^, survival^47^ and survminer^48^ packages.

A 5-fold cross validation was performed: the original dataset was randomly partitioned into 5 equal sized subsamples. Of the 5 subsamples, a single subsample was retained as the validation data for testing the model, and the remaining 4 subsamples were used as training data. The cross-validation process was then repeated 5 times, with each of the 5 subsamples used once as the validation data. Values are reported as a mean of the 5 models. To limit test-set leakage, we calculated the ICC and the Wilcoxon correlation in 4 of the 5 partitioned datasets (that were created for the 5-fold cross-validation), leaving out the validation set from the first validation.

### Deep learning training and validation

Robust features significantly correlated to pCR were used as inputs to a Deep Neural Network (DNN) created using the DNNClassifier Custom Estimator from the TensorFlow open-source framework (v1.3, Google, Mountain View, California, USA)^49^. We explored a range of combinations of batch size, layer depth and layer size. We determined the optimal architecture for this deep learning model empirically, testing numerous variants. Changing the depth of the network reduced performance. We did not increase our model depth beyond 10,000 hidden units due to computational constraints. The resulting DNN was a compromise between performance and computational cost and included three hidden layers with 10, 20 and 10 neurons respectively. A Rectified Linear Unit (ReLu) function^50^ was used for activation in the hidden layers because it resulted in a faster training: $F(x)=\,\max (x,\,0)$, where x is the input to a neuron. Gradient descent was performed with the Adagrad Optimizer (equation 2)^51^:

2 $${\rm{\Theta }}t+1={\rm{\Theta }}t-\frac{\eta }{\sqrt{Gt+\,\varepsilon }}\,\ast \,gt$$

where ${\rm{\Theta }}$ are parameters, t is the time-step, $\eta$ is the learning rate, gt is the gradient, Gt is a matrix of the sum of the squares of gradients up to time step t, and $\varepsilon$ is a smoothing term that avoids division by zero. Adagrad adapts the learning rate to the parameters, performing larger updates for infrequent and smaller updates for frequent parameters. For this reason, it is well-suited for sparse data. The output of the network was binary (pCR or no pCR).

To avoid overfitting, a low number of epoch was chosen (3000 steps, 1 epoch). Training and validation was performed on a Linux Ubuntu 17.04 workstation with a Quad Core 2.8 Ghz Intel Core i7-770HQ and a GeForce GTX1060 Graphics Processing Unit (GPU). Results were visualized with the TensorBoard suite (Google, Mountain View, California, USA).

A logistic regression model was built from the same training and testing datasets using only the TNM stage as a baseline comparison, with the glmnet R package^52^. A Support Vector Machine model was created with the same variables as the DNNClassifier with Sci-Kit Learn^53^. AUCs were calculated with the pROC R package^39,54^.

**Figure 3:**
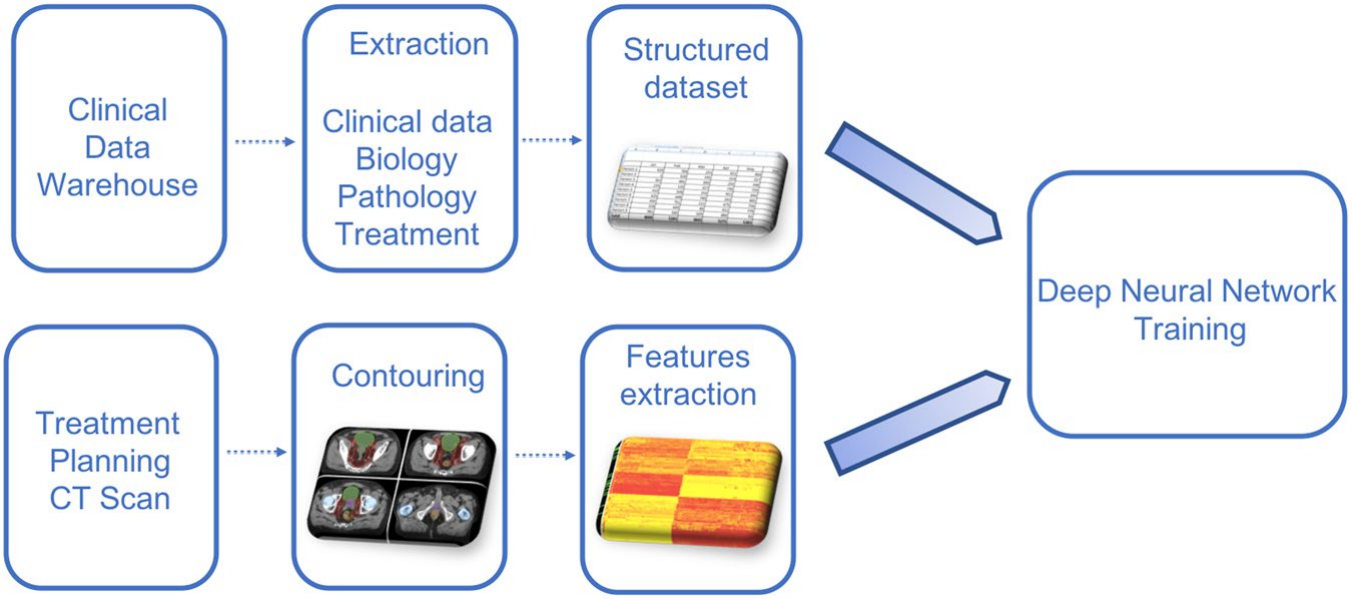
Global analysis pipeline.

The global analysis pipeline is shown in Fig. 3.

## Electronic supplementary material

Supplementary file

## Data Availability

The datasets generated during and/or analyzed during the current study are not publicly available due to the clinical and confidential nature of the material but can be made available from the corresponding author on reasonable request.
