# Author Correction: Deep Learning and Radiomics predict complete response after neo-adjuvant chemoradiation for locally advanced rectal cancer

**DOI:** 10.1038/s41598-018-35359-7

**Published:** 2018-11-12

**Authors:** Jean-Emmanuel Bibault, Philippe Giraud, Martin Housset, Catherine Durdux, Julien Taieb, Anne Berger, Romain Coriat, Stanislas Chaussade, Bertrand Dousset, Bernard Nordlinger, Anita Burgun

**Affiliations:** 1 Radiation Oncology Department, Georges Pompidou European Hospital, Assistance Publique – Hôpitaux de Paris, Paris Descartes University, Paris Sorbonne Cité, Paris, France; 2 INSERM UMR 1138 Team 22: Information Sciences to support Personalized Medicine, Paris Descartes University, Sorbonne Paris Cité, Paris, France; 3 Department of gastroenterology and digestive oncology, Georges Pompidou European Hospital, Assistance Publique – Hôpitaux de Paris, Paris Descartes University, Paris Sorbonne Cité, Paris, France; 4 Department of General Surgery and Surgical Oncology, Georges Pompidou European Hospital, Assistance Publique – Hôpitaux de Paris, Paris Descartes University, Paris Sorbonne Cité, Paris, France; 5 Gastroenterology and digestive oncology unit, Cochin University Hospital, Assistance Publique – Hôpitaux de Paris, Paris, France; 6 INSERM Y 1016, Université Paris Descartes, Paris, France; 7 Department of Digestive, Hepato-biliary and Endocrine Surgery, Cochin Hospital, Assistance Publique –Hôpitaux de Paris, Paris, France; 8 Department of General Surgery and Surgical Oncology, Hôpital Ambroise Paré, Assistance Publique – Hôpitaux de Paris, Boulogne-Billancourt, France; 9 Biomedical Informatics and Public Health Department, Georges Pompidou European Hospital, Assistance Publique – Hôpitaux de Paris, Paris Descartes University, Paris Sorbonne Cité, Paris, France

Correction to: *Scientific Reports* 10.1038/s41598-018-30657-6, published online 22 August 2018

Martin Housset was omitted from the author list in the original version of this Article. This has been corrected in the PDF and HTML versions of the Article, and in the accompanying Supplementary Information file.

The Author Contributions section now reads:

J.E.B. conceived and developed the research idea with the assistance of P.G. and A.B. The study design was performed by J.E.B., P.G. and A.B. J.E.B., M.H. and C.D. performed the segmentation task. J.E.B., M.H., C.D., J.T., A.B., B.D., R.C., S.C., B.N. performed data collection. J.E.B. performed the computational experiments with guidance from P.G., A.B. The paper was written by J.E.B. with technical content from A.B., and extensive editorial input from all authors.

