# Supplementary material for: Deep Learning and Radiomics predict complete response after neo-adjuvant chemoradiation for locally advanced rectal cancer
Source: Sci Rep. 2018 Aug 22;8:12611. doi: 10.1038/s41598-018-30657-6 (PMC6105676; doi:10.1038/s41598-018-30657-6)
Supplement: Supplementary file 1 — Supplementary file [file 41598_2018_30657_MOESM1_ESM.docx]

**Supplementary file**

Deep Learning and Radiomics predict complete response after neo-adjuvant chemoradiation for locally advanced rectal cancer

Jean-Emmanuel Bibault, Philippe Giraud, Martin Housset, Catherine Durdux, Julien Taieb, Anne Berger, Romain Coriat, Stanislas Chaussade, Bertrand Dousset, Bernard Nordlinger, Anita Burgun

| **Category** | **Feature algorithm** |
| --- | --- |
| Shape | SurfaceArea  SurfaceAreaDensity  Mass  Convex  ConvexHullVolume  ConvexHullVolume3D  MeanBreath  Orientation  Roundness  NumberOfObjects  NumberOfVoxel  VoxelSize |
| Intensity Direct | Skewness  Range  Percentile  Quantile  InterQuartileRange  GlobalEntropy  GlobalUniformity  GlobalMax  GlobalMin  GlobalMean  GlobalMedian  GlobalStd  MeanAbsoluteDeviation  MedianAbsoluteDeviation  LocalEntropy/Range/StdMax  LocalEntropy/Range/StdMin  LocalEntropy/Range/StdMean  LocalEntropy/Range/StdMedian  LocalEntropy/Range/StdStd |
| Gray Level Coocurrence Matrix 25  Gray Level Coocurrence Matrix 3 | Contrast  Correlation  Energy  Homogeneity |
| Neighbor Intensity Difference 25 Neighbor Intensity Difference 3 | Busyness  Coarseness  Complexity  Contrast  TextureStrength |
| Gray Level Run Length Matrix 25 Gray Level Run Length Matrix 3 | GrayLevelNonuniformity  HighGrayLevelRunEmphasis  LongRunEmphasissis  LongRunHighGrayLevelEmphasis  LongRunLowGrayLevelEmphasis  LowGrayLevelRunEmphasis  RunLengthNonuniformity  RunPercentage  ShortRunEmphasissis  ShortRunHighGrayLevelEmphasis  ShortRunLowGrayLevelEmphasis |

**Supplementary File 1.** Radiomics features categories extracted from the CT Scans. 25: Features are computed from all 2D image slices; 3: Features are computed from 3D image matrix
